# Overexpression of *OsMBL1* Is Associated with Changes in Flavonoid Biosynthesis and Antioxidant Capacity in Rice

**DOI:** 10.3390/biology15161378

**Published:** 2026-08-12

**Authors:** Menghan Zhu, Zhongwen Zhan, Fan Fei, Yuxing Cai, Ming Ding, Haidong Ding

**Affiliations:** College of Bioscience and Biotechnology, Yangzhou University, Yangzhou 225009, China

**Keywords:** *OsMBL1*, transcriptome, flavonoids, antioxidant, stress tolerance

## Abstract

Plant-derived lectins originating from plants perform essential functions in both plant growth and stress response processes. Rice jacalin-related mannose-binding lectin 1 (OsMBL1), a well-characterized canonical salt-responsive regulatory factor, acts as a core positive regulator that confers enhanced salt tolerance. In the present study, *OsMBL1* was found to respond to multiple environmental stresses and signaling molecules, and it not only positively regulated salt tolerance at the seedling stage but also at the seed germination stage. To dissect the underlying regulatory network, we performed transcriptome profiling of an *OsMBL1*-overexpressing line, which identified 562 differentially expressed genes (438 up- and 124 down-regulated) relative to wild-type plants. These sets of differentially expressed genes exert pivotal control over a suite of physiological events, most notably the conserved metabolic routes of phenylpropanoid and flavonoid biosynthesis. Further physiological assays confirmed a low level of membrane lipid peroxidation and a high level of flavonoids and ascorbate peroxidase (APX) and peroxidase antioxidant enzyme activities in the *OsMBL1*-overexpressing line. Collectively, these correlative findings suggest that *OsMBL1* overexpression is associated with the upregulation of genes involved in flavonoid biosynthesis and with an altered antioxidant system status, which may contribute to enhanced stress tolerance in rice. Collectively, these observations provide a deeper perspective on the molecular underpinnings of OsMBL1-associated stress adaptation.

## Simple Summary

This study shows that a rice protein, OsMBL1, can significantly boost salt tolerance. When *OsMBL1* was overexpressed, rice seeds germinated better and seedlings grew stronger under salt stress. By comparing gene activity, it was found that *OsMBL1* is associated with increased production of flavonoids and higher activities of antioxidant enzymes like APX and peroxidase. These changes reduce harmful lipid damage and keep cells healthier. Our findings suggest that OsMBL1 may act as part of a regulatory network, turning on defense genes to protect rice from stress.

## 1. Introduction

Lectins, functional proteins widely distributed in nature, specifically and reversibly bind carbohydrates and serve as core molecular recognition carriers for the sensing and decoding of glycosyl signals through the precise recognition of biological information encoded by glycosyl modifications [1]. To date, many plant-derived lectins have been both identified and assigned functional roles; they participate in a wide range of biological processes including plant development, immunity, stress signal transduction, and gene expression regulation [2]. However, further systematic investigations are still required to elucidate the detailed molecular mechanisms whereby plant lectins mediate stress tolerance [3].

Jacalin-related lectins (JRLs) are carbohydrate-recognition proteins with a ubiquitous occurrence across plant species. They exert pivotal functions in plant development and pathogen defense [4]. As one crucial member of this gene family, *OsJRL40* has been demonstrated to positively modulate salt stress tolerance in rice [5]. In addition to the regulatory function of JRL members in abiotic stress adaptation such as salt tolerance, another jacalin-related lectin gene, *OsJacLK1*, has been proven to exert a positive regulatory effect on rice resistance against the devastating fungal pathogen *Magnaporthe oryzae* [6]. *OsMBL1*, also named *OsSalT* and *Osl43*, encodes a 145-amino-acid polypeptide classified as a jacalin-type mannose-binding lectin, which exerts indispensable biological roles in rice responses to diverse adverse conditions including salinity, drought and cold stress, as well as host immune defense against pathogenic infection [4,7,8,9]. Furthermore, *OsMBL1* has also been demonstrated to participate in the regulation of rice leaf senescence [10,11]. Nevertheless, several critical molecular questions surrounding the regulatory mechanism of OsMBL1 remain insufficiently clarified and await further in-depth exploration.

In plants, flavonoids count as one of the predominant polyphenolic secondary metabolites in terms of abundance and have gained increasing recognition for their significance in biosynthesis and regulatory responses to environmental stress [12]. Flavonoids, through their robust antioxidant action, effectively alleviate oxidative damage, thereby safeguarding pollen viability, seed development, and overall plant fitness [12,13]. Recently, a report showed that OsJRL can regulate rice cold tolerance via inhibiting phenylalanine metabolism and flavonoid biosynthesis [9]. Nevertheless, despite the well-established physiological functions of flavonoids, the underlying molecular regulatory networks remain largely elusive [14,15].

Our previous studies have revealed that OsMBL1 exerts a positive regulatory effect on salt tolerance in rice at the seedling stage and is regulated by the rice RING E3 ligase OsRMT1 [16]; however, the underlying mechanism of OsMBL1 remains to be further elucidated. In this study, we further found that *OsMBL1* responds to multiple environmental stresses and signaling molecules, and it also positively regulates salt tolerance at the seed germination stage. Further transcriptome sequencing analysis indicated that *OsMBL1* is associated with the altered expression of key genes involved in phenylalanine metabolism and flavonoid biosynthesis. In addition, the degree of membrane lipid peroxidation was decreased, and the activity of key antioxidant enzymes was increased. In all, these observations provide a deeper perspective on the molecular regulatory mechanism underlying OsMBL1-mediated stress tolerance.

## 2. Materials and Methods

### 2.1. Plant Materials and Stress Treatments

Plump seeds of upland rice cultivar Zhonghan3 were selected and soaked in sterile distilled water for 24 h and then placed on moist filter papers for germination. The construction of *OsMBL1* overexpression lines and salt tolerance evaluation of rice in this study were carried out as in our previous report [16]. When the seedlings grew to the three-leaf stage, robust and consistently grown rice plants were separately treated with 150 mM NaCl, 10% polyethylene glycol (PEG), 50 μM abscisic acid (ABA), 10 mM hydrogen peroxide (H_2_O_2_), 100 μM sodium nitroprusside (SNP), and 10 mM calcium chloride (CaCl_2_). Rice seedlings cultured in a normal nutrient solution were set as blank controls. Leaf samples were collected at 0.5, 1, 6, and 12 h after treatment. After rapid freezing in liquid nitrogen, all samples were maintained at −80 °C for later use.

### 2.2. RNA Extraction and qRT-PCR

Plant RNA was extracted with the RNA isolater reagent (Vazyme Biotech, Nanjing, China) according to the manufacturer’s guidelines. After quality assessment by measuring purity and concentration, 1 μg of the total RNA was subjected to reverse transcription using the HiScript II kit (Vazyme) to obtain first-strand cDNA. The qRT-PCR assay was carried out with AceQ Universal SYBR Master Mix (Vazyme) on a Step One Plus instrument (ABI Step One-Plus, Foster City, CA, USA), where *OsUBQ* was chosen as the reference gene. For each biological sample, PCR amplifications were performed in triplicate (technical replicates) with the oligonucleotides listed in Supplementary Table S1.

### 2.3. Seed Germination Assay

Uniform and plump seeds of wild-type (WT) and transgenic rice were selected and soaked in NaCl solutions with concentrations of 0, 100, 150, and 200 mM at 28 °C for 24 h. Sterile Petri dishes were prepared with two layers of sterilized filter paper laid at the bottom. The soaked seeds were evenly placed in the dishes, and 20 mL of corresponding treatment solution was added to each dish before labeling. All samples were incubated in a light incubator at 28 °C with a photoperiod of 12 h light/12 h dark. Seed germination status was counted at regular intervals, and radicle protrusion was defined as the germination standard.

### 2.4. RNA-Seq Analysis

The OE-118 line, which exhibited the most pronounced salt-tolerant phenotype in our preliminary screening [16], was selected for transcriptomic analysis to maximize the detection of gene expression changes associated with *OsMBL1* overexpression. Under normal culture conditions, leaves at the same leaf position with uniform growth status were collected from 4-week-old WT plants and the *OsMBL1*-overexpressing line (OE-118). Three biological replicates were included per sample group, and all collected materials were rapidly frozen in liquid nitrogen and kept at −80 °C. Subsequent RNA-seq was performed by Shanghai Majorbio Bio-Pharm Technology Co., Ltd. (Shanghai, China), using the same set of samples, generating 150 bp paired-end reads. After quality filtering, clean reads were aligned to the rice reference genome (Rice genome assembly IRGSP-1.0, MSU v7.0) using HISAT2 v2.1.0. Gene expression levels were quantified, and differential expression analysis was performed using DESeq2 v1.24.0. Genes with |log_2_(fold change)| ≥ 1 and false discovery rate (FDR) < 0.05 were considered differentially expressed.

### 2.5. Physiological Index Measurements

0.5 g of pre-frozen leaf tissue was fully homogenized in 10 mL of ice-cold extraction buffer, including 50 mM phosphate (pH 7.0), 1 mM EDTA, and 1% (*w*/*v*) PVP. The resulting homogenate was centrifuged at 5000× *g* for 20 min under 4 °C, and the clarified supernatant, defined as crude enzyme extract, was collected for subsequent biochemical assays. Peroxidase (POD) activity was assayed using guaiacol as the reaction substrate, by continuously monitoring the absorbance increment at 470 nm (molar extinction coefficient = 26.6 mM^−1^·cm^−1^). Ascorbate peroxidase (APX) activity was measured by recording the absorbance decrease at 290 nm caused by AsA oxidation, using the extinction coefficient of 2.8 mM^−1^·cm^−1^ for calculation. Lipid peroxidation level was estimated by measuring thiobarbituric acid reactive substances (TBARS), a widely used indicator of oxidative stress, according to the method described by Ding et al. [17] with minor modifications. It is important to note that this assay detects TBARS rather than malondialdehyde (MDA) specifically, as the TBA reaction may be influenced by other compounds in plant tissues. Determination of total flavonoids was accomplished by a slightly modified version of the Herald et al. [18] procedure, with subsequent quantification performed against the standard curve.

### 2.6. Statistical Analysis

Each physiological and qRT-PCR experiment was conducted with at least three independent biological replicates, with each biological replicate consisting of three technical replicates for qRT-PCR. Data are presented as mean ± standard deviation (SD) of the biological replicates. Depending on the comparison design, we used either one-way ANOVA plus Tukey’s post hoc test (for multiple groups) or Student’s *t*-test (for two groups) to evaluate intergroup differences.

## 3. Results

### 3.1. Expression Patterns of OsMBL1

To clarify the response characteristics of *OsMBL1* induced by abiotic stresses, phytohormones and signaling molecules, the expression changes in this gene were detected by qRT-PCR. After exposure to 150 mM NaCl, 10% PEG, 50 μM ABA and 10 mM H_2_O_2_, the transcript abundance of *OsMBL1* was markedly elevated and gradually accumulated to the highest level at 12 h. In comparison, treatment with 100 μM SNP and 10 mM CaCl_2_ triggered an earlier expression peak, and the transcriptional level of *OsMBL1* reached its maximum at 6 h (Figure S1). Collectively, these findings strongly indicate that *OsMBL1* participates in the regulatory network of stress adaptation and may serve as an important functional gene involved in plant abiotic stress resistance.

### 3.2. Overexpression of OsMBL1 Promotes Salt Tolerance

**Figure 1:**
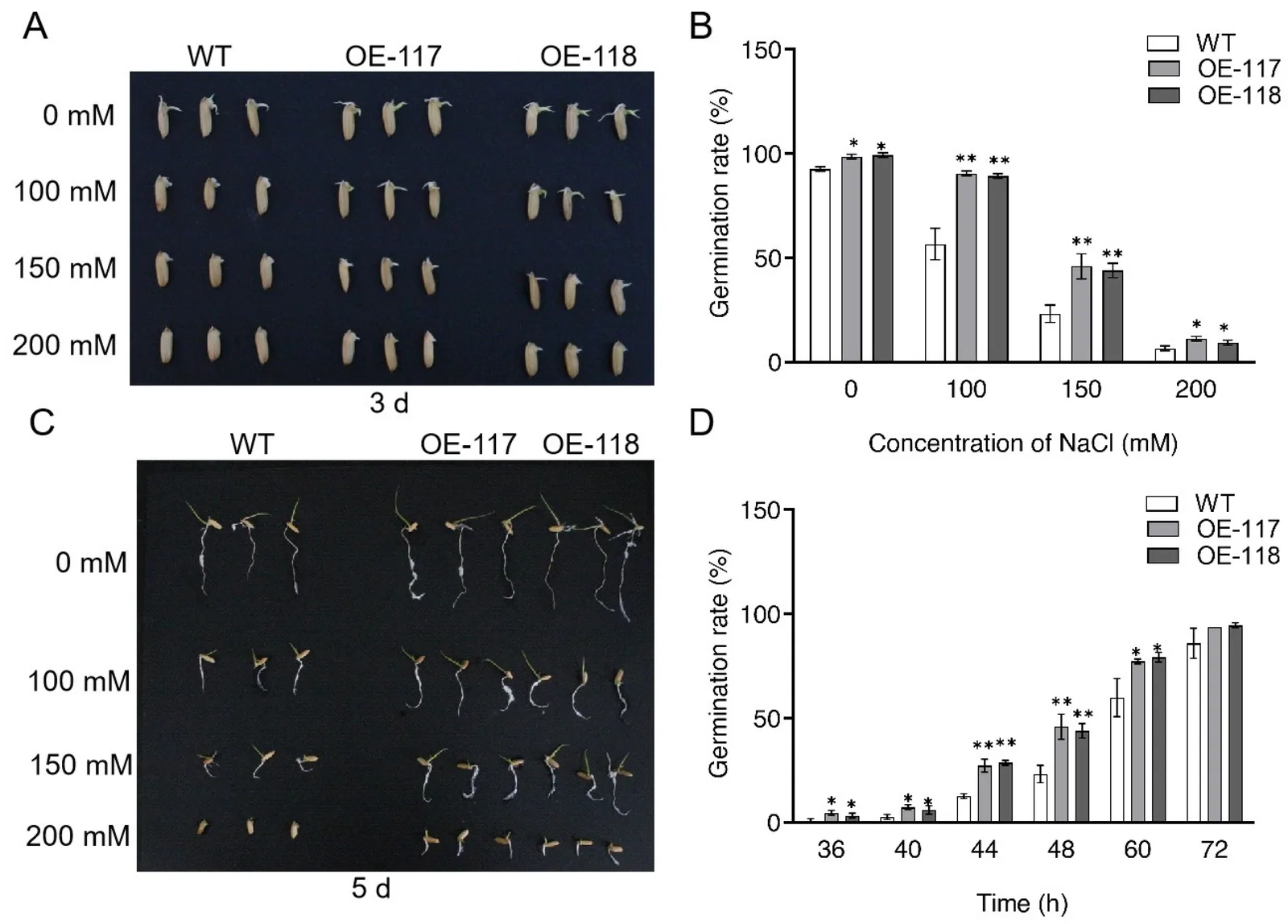
Effects of NaCl treatment on seed germination of WT and *OsMBL1*-overexpressing plants. (**A**,**C**) Phenotypes of shoot and root growth in WT and *OsMBL1*-overexpressing plants after exposure to a concentration series of NaCl (0, 100, 150, and 200 mM) for 3 and 5 days. (**B**) Germination rates of WT and *OsMBL1*-overexpressing plants at 48 h under different NaCl concentrations. (**D**) Dynamic changes in the germination rates of WT and *OsMBL1*-overexpressing plants under 150 mM NaCl treatment. Data are mean ± SD (n ≥ 3). Different asterisks indicate significant differences determined by Student’s *t*-test compared with WT (* *p* < 0.05; ** *p* < 0.01).

To evaluate the impact of *OsMBL1* on seed germination under salt conditions, seeds were subjected to a concentration series of NaCl (0, 100, 150, and 200 mM), and germination phenotypes were observed after 3 and 5 days of incubation. The results showed that the seed germination rate of *OsMBL1* overexpression lines was significantly higher than that of WT at 3 days of salt treatment. The seed germination efficiency of *OsMBL1* overexpression lines was obviously higher (Figure 1A,B). Moreover, the seedling shoot and root lengths of overexpression lines were notably longer than those of WT at 5 days after germination (Figure 1C). The overexpression lines possessed superior seed germination performance (Figure 1D). These findings suggested that *OsMBL1* can effectively facilitate rice seed germination and distinctly improve seed germination capacity and salt tolerance at the germination stage under saline conditions.

The growth of salt tolerance in rice was further investigated (Figure S2A). After treatment with 150 mM NaCl for 10 days, more green surviving seedlings were retained in OsMBL1 overexpression lines (Figure S2B). Following recovery cultivation for 7 days, the overexpression lines exhibited better growth status and markedly higher survival rates than WT plants (Figure S2C,D), which further confirmed that overexpression of OsMBL1 enhances salt stress tolerance in rice.

### 3.3. OsMBL1 Overexpression Is Associated with Altered Expression of Phenylpropanoid and Flavonoid Biosynthesis Genes in Rice

**Figure 2:**
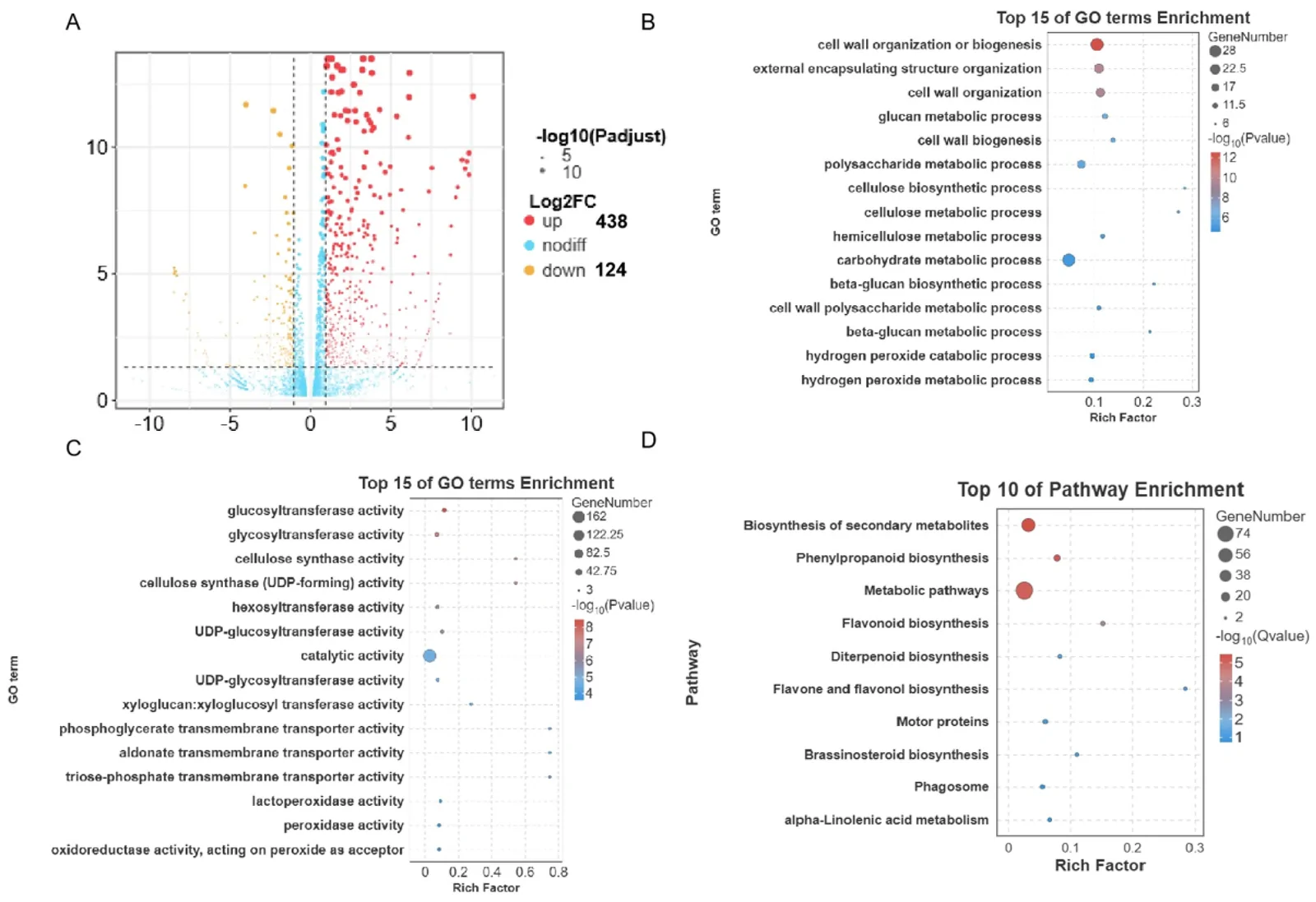
Transcriptome analyses of WT and *OsMBL1*-OE line (OE-118) under normal conditions. (**A**) Volcano plot of differentially expressed genes between WT and *OsMBL1*-overexpressing plants under normal conditions. Red dots indicate upregulated genes, and yellow dots indicate downregulated genes. (**B**,**C**) GO enrichment analysis of common differentially expressed genes between WT and *OsMBL1*-overexpressing plants under normal conditions demonstrated the regulatory role of *OsMBL1* in biological process and molecular function, respectively. The top 15 and 10 enriched terms are shown, respectively. The top 15 enriched terms are shown. (**D**) KEGG analysis of common differentially expressed genes between WT and *OsMBL1*-overexpressing plants under normal conditions. The top 10 enriched terms are shown.

To explore the genes and pathways whose expression correlates with *OsMBL1* overexpression, transcriptome sequencing was performed on leaf tissues of WT and *OsMBL1*-overexpressing rice grown under normal conditions. Differential gene analysis identified a total of 562 differentially expressed genes (DEGs) in *OsMBL1*-OE relative to WT, among which 438 were upregulated and 124 were downregulated (Figure 2A, Table S2). Biological Process enrichment revealed that DEGs were predominantly enriched in external encapsulating structure organization, cell wall organization or biogenesis, cell wall organization, carbohydrate metabolic processes, etc. (Figure 2B, Table S3). Molecular Function enrichment showed that most DEGs were annotated to glucosyltransferase activity, cellulose synthase activity, hexosyltransferase activity, UDP-glucosyltransferase activity, catalytic activity, etc. (Figure 2C, Table S3). KEGG enrichment indicated that the DEGs were significantly associated with biosynthesis of secondary metabolites, phenylpropanoid biosynthesis, metabolic pathways and flavonoid biosynthesis (Figure 2D, Table S4). Collectively, these data, obtained under normal growth conditions, indicate that *OsMBL1* overexpression is correlated with a profound remodeling of the transcriptome, particularly affecting genes involved in cell wall formation and secondary metabolism, including the phenylpropanoid and flavonoid biosynthetic pathways. These transcriptional changes may establish a primed state that could contribute to enhanced stress tolerance, although this hypothesis requires direct testing under stress conditions.

### 3.4. Validation of Key Pathway Gene Expression

**Figure 3:**
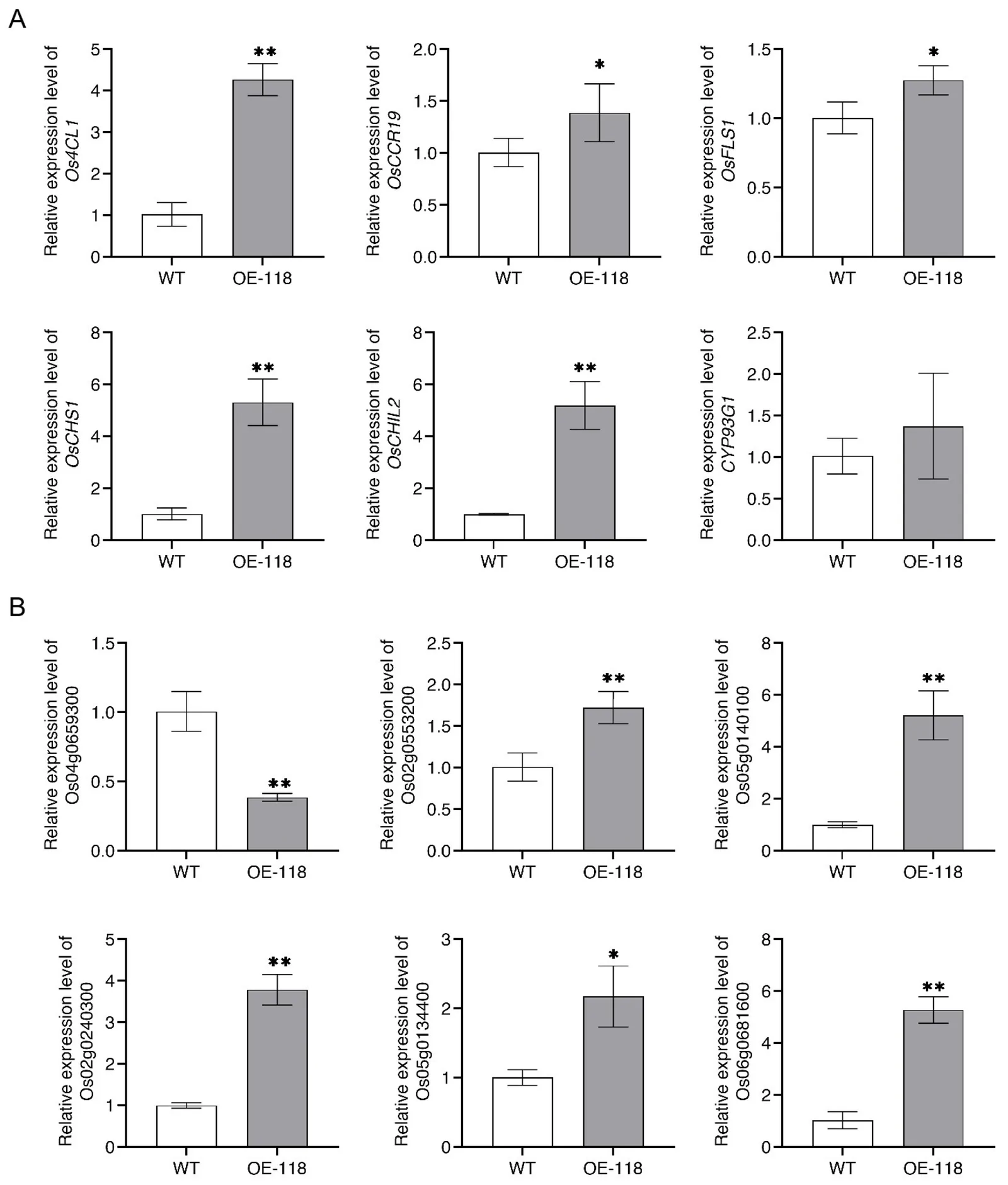
Validation of transcriptomic differentially expressed genes by qRT-PCR. (**A**) Verification of the expression levels of genes involved in the phenylalanine and flavonoid biosynthesis by qRT-PCR. (**B**) Verification of the expression levels of genes involved in the reactive oxygen species metabolic process by qRT-PCR. Data are presented as mean ± SD (n = 3). Different asterisks indicate significant differences based on Student’s *t*-test (* *p* < 0.05; ** *p* < 0.01).

Next, we further analyzed the transcript levels of genes involved in phenylpropanoid and flavonoid biosynthesis in leaves of WT and *OsMBL1*-OE plants under normal conditions using qRT-PCR. Multiple key genes of these two pathways, such as *Os4CL1* (Os08g0245200), *OsCCR19* (Os09g0419200), *OsCHS1* (Os11g0530600), *OsCHIL2* (Os12g0115700), and *OsCYP93G1* (Os04g0101400), were significantly upregulated in *OsMBL1*-OE rice (Figure 3A). In addition, we verified the expression patterns of genes related to stress tolerance and the ROS metabolic process, such as Os04g0659300 (*OsRMC*), Os02g0553200 (*OsAPx8*), Os05g0140100 (*OsMYB61L*), Os02g0240300 (prx), Os05g0134400 (prx), and Os06g0681600 (prx). The consistency between the trends and the transcriptome profiles served to validate the quality of the RNA-seq results (Figure 3B). Taken together, these qRT-PCR data corroborate the transcriptomic findings, showing that *OsMBL1* overexpression positively correlates with the expression of phenylpropanoid and flavonoid biosynthetic genes under normal conditions, which are speculated to enhance salt tolerance in rice. Future metabolomic profiling is required to identify the exact flavonoid species responsible for the antioxidant effects.

### 3.5. Effects of Overexpressing OsMBL1 Gene on Plant Antioxidant System

**Figure 4:**
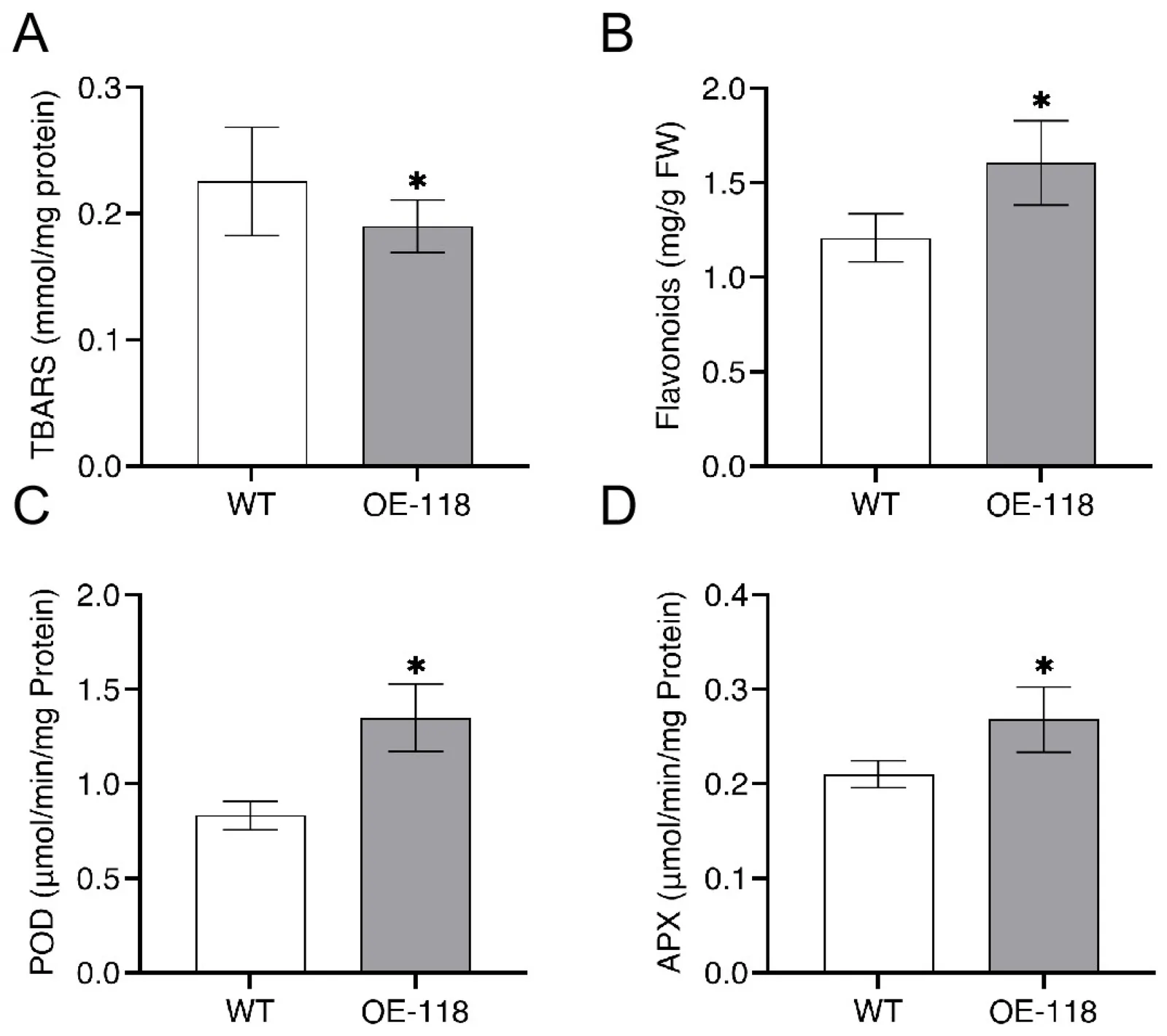
Effects of overexpressing *OsMBL1* gene on plant antioxidant system exposed to salt stress. (**A**) TBARS level. (**B**) Flavonoid content. (**C**) POD activity. (**D**) APX activity. Data are presented as mean ± SD (n = 3). Different asterisks indicate significant differences based on Student’s *t*-test compared with WT (* *p* < 0.05).

**Figure 5:**
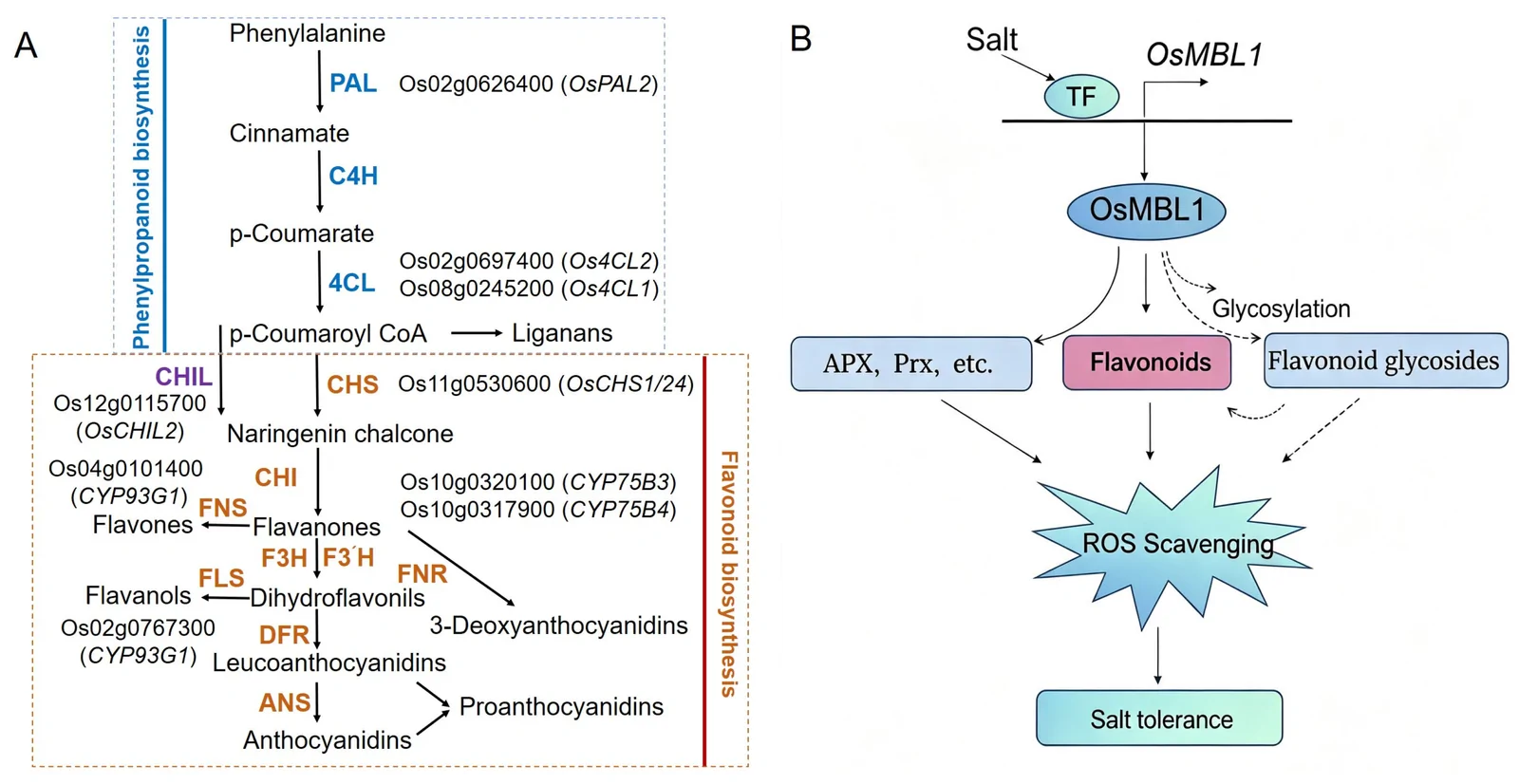
A proposed working model illustrating the potential link between *OsMBL1* overexpression, flavonoid metabolism, and salt tolerance in rice. (**A**) Simplified flavonoid biosynthesis pathway in plants. The entire pathway comprises two sequential parts: the early phenylpropanoid module and the dedicated flavonoid branch. The rate-limiting enzymes at each step are highlighted in color. The abbreviated enzyme names are as follows: PAL (phenylalanine ammonia-lyase), C4H (cinnamic acid 4-hydroxylase), 4CL (4-coumarate-CoA ligase), CHS (chalcone synthase), CHI (chalcone isomerase), CHIL (chalcone isomerase-like), FNS (flavone synthase), F3H (flavanone 3-hydroxylase), FLS (flavonol synthase), F3′H (flavonoid 3′-hydroxylase), DFR (dihydroflavonol 4-reductase), ANS (anthocyanidin synthase), and FNR (flavanone 4-reductase). (**B**) A hypothetical model of OsMBL1-mediated salt tolerance. This model is inferred from transcriptomic and physiological data obtained from an *OsMBL1* overexpression line under non-stress conditions and is intended to guide future investigations. Solid arrows represent experimentally supported associations, whereas dashed arrows indicate inferred or speculative relationships that await direct validation.

Seedlings of WT and *OsMBL1*-overexpressing lines were grown to the two-leaf-and-one-heart stage, and TBARS levels, flavonoid accumulation, and the activities of POD and APX, two representative antioxidant enzymes, were assayed in leaf samples under normal conditions (Figure 4). TBARS are essentially a class of highly reactive three-carbon dialdehydes and are the most widely used characteristic biomarkers in the current field of oxidative stress research. Leaves of *OsMBL1*-overexpressing rice displayed a lower level of TBARS (Figure 4A). The results revealed that the *OsMBL1*-overexpressing line exhibited higher levels of total flavonoids (Figure 4B) and antioxidant enzyme activities (Figure 4C,D). These physiological changes indicate an altered antioxidant status in the overexpression line, characterized by reduced lipid peroxidation markers and increased activities of key ROS-scavenging enzymes. Collectively, these results indicate that *OsMBL1* overexpression is associated with an enhanced antioxidant status in rice (Figure 5)**,** but this interpretation requires direct validation through ROS measurements in future studies.

## 4. Discussion

Transcription of *OsMBL1* was markedly induced by treatments with NaCl, PEG, ABA, H_2_O_2_, SNP, and CaCl_2_ (Figure S1), indicating that multiple signaling molecules are involved in the regulation of the expression of this gene. Considering that *OsMBL1* is strongly induced by ABA at the transcriptional level (Figure S1), we hypothesize that *OsMBL1* participates in salt stress responses through ABA-dependent pathways, yet in-depth mechanistic research on this process in rice is still limited. Despite this limitation, cumulative research evidence identifies *OsMBL1* as a pivotal gene governing salt stress, drought stress responses, and ABA signal transduction [4,7]. Accumulating studies have demonstrated that *OsMBL1* acts as a positive regulator of rice seedling salt tolerance [19]. Here, the results further confirmed that overexpression of *OsMBL1* substantially improves salt tolerance at the rice germination stage and seedling stage (Figure 1 and Figure S2). The RING-type E3 ubiquitin ligase OsRMT1 ubiquitinates OsMBL1 and facilitates its degradation, thereby negatively modulating rice salt tolerance [19]. Moreover, OsMBL1 serves as a ubiquitination substrate of RING-type E3 ubiquitin ligase OsSIRH2-23, which also mediates the degradation of OsMBL1 [20]. These findings further indicate that OsMBL1 is tightly regulated via post-translational ubiquitination modification during stress responses. Functional characterization also uncovered that *OsMBL1* positively regulates drought resistance and immune responses [4,8,21,22], yet negatively modulates cold tolerance in rice [9].

What is the mechanism of *OsMBL1* in regulating multiple stresses? Our transcriptomic data from the overexpression line grown under normal conditions demonstrate that OsMBL1 overexpression is correlated with altered expression of genes associated with phenylpropanoid and flavonoid biosynthesis in rice (Figure 2). It is important to emphasize that these transcriptional changes were observed constitutively, in the absence of salt stress, and therefore represent a pre-activated transcriptional state rather than a stress-induced dynamic response. The causal relationship between these constitutive changes and the improved salt tolerance phenotype remains to be established through time-course transcriptomic analysis under salt stress conditions. Here we selected OE-118 because it showed the highest expression level and most consistent salt-tolerant phenotype in our preliminary screening, making it a representative line for transcriptomic analysis [16]. The phenylpropanoid pathway represents a core secondary metabolic route in plants, whose downstream flavonoid metabolites function as one of the major antioxidants, serving as vital defensive compounds for plant adaptation to diverse abiotic challenges [12,14]. Flavonoid biosynthesis is a multi-step process governed by a set of critical enzymes, among which phenylalanine ammonia-lyase (PAL) and 4-coumarate-CoA ligase (4CL) play the gateway roles. Specifically, PAL initiates the pathway by removing the amino group from phenylalanine, generating cinnamic acid, while 4CL subsequently activates the acid to its coenzyme A ester, cinnamoyl-CoA. This study found that one PAL (*OsPAL2*) and two 4CLs (*Os4CL1* and *Os4CL2*) were upregulated in the *OsMBL1*-overexpression line. The five 4CL genes in the rice genome exhibit no obvious tissue-specific expression patterns; however, their expression levels differ markedly [13]. The initial steps of flavonoid biosynthesis are catalyzed by chalcone synthase (CHS), a key enzyme. Chalcone isomerase (CHI) enhances the isomerization efficiency from chalcone to flavanone to a great extent, and additionally constitutes a critical rate-limiting determinant of the pathway. Flavone synthase (FNS) is the principal enzyme responsible for the biosynthesis of flavones. Here, the expression of *OsCHS24* (*OsCHS1*) and *CYP93G1* was also higher in the *OsMBL1*-overexpression line. OsCHS24 catalyzes the condensation of p-coumaroyl-CoA and malonyl-CoA to form naringenin chalcone. With higher expression and stronger enzymatic activity, OsCHS24 is the major chalcone synthase isozyme in rice. In the leaves of the *oschs24* mutant, the expression of *OsCHS24* was greatly suppressed after UV irradiation, and the content of sakuranetin decreased [23]. CHI, the second key enzyme of flavonoid biosynthesis, isomerizes naringenin chalcone to naringenin stereospecifically. Stress-driven OsCHIL2 expression raises flavonoid content and reinforces rice tolerance to environmental stresses [24]. CYP93G1 is a key branch-point enzyme in rice that converts flavanones into O-methylflavone conjugates, catalyzing the desaturation of naringenin and eriodictyol to apigenin and luteolin, respectively [25]. CYP75B4 functions as a bona fide flavonoid 3′-hydroxylase. In vitro, CYP75B4 catalyzes the 3′-hydroxylation of various flavonoids. CYP75B3 is co-expressed with OsF2H and serves as the sole F3′H in the biosynthesis of flavone C-glycosides [26].

Flavonoid glycosides with complex structures are key phytochemicals for plant stress defense. Glycosyltransferase-mediated glycosylation regulates the stability, accessibility, and activity of flavonoids, including anthocyanins [15,27]. In this study, GO analysis revealed that a large number of genes involved in glycosyltransferase activity show altered expression in the *OsMBL1*-overexpression line, such as UGT88C3 (Os07g0510500), OsUGT90A1 (Os04g0305700), and UGT707A3 (Os07g0503500). Glycosyltransferases have been implicated in plant stress adaptation through their effects on flavonoid metabolism. For instance, rice OsDUGT1 functions in heat tolerance via flavonoid glycosylation and metabolic regulation [15], whereas GSA1 (UGT83A1) orchestrates the diversion of precursors from lignin to flavonoid pathways under stress, enabling the production of glycosylated flavonoids that serve as stress protectants [27]. In rice, the overexpression of *OsUGT90A1* improves salt tolerance, a phenotype linked to heightened antioxidant enzyme activities [28]. In addition, we found that the expression of a transcription factor, *OsMYB2P-1* (Os05g0140100), is also higher in the *OsMBL1*-overexpression line. OsMYB2P-1 is an R2R3 MYB-type transcription factor and can directly bind the *UGT706F1* promoter and trigger its transcription. Overexpression of this gene further boosts heat tolerance and flavonoid glycoside production in rice [29]. In addition, the genes encoding OsAPx8 and multiple Prxs were found to be upregulated, suggesting that their increased expression may play important roles in the altered antioxidant enzyme activities observed in the overexpression line. It is important to note that our transcriptomic analysis was conducted under normal growth conditions, which captured the constitutive transcriptional reprogramming driven by *OsMBL1* overexpression. While this approach revealed key metabolic pathways that are pre-activated, it does not fully capture the dynamic transcriptional response to salt stress itself. Future studies employing stress-time-course RNA-seq will be necessary to distinguish between direct and secondary regulatory effects of *OsMBL1* during salt challenge. While our data strongly support a positive correlation between *OsMBL1* abundance and tolerance, the definitive biological function of the endogenous gene will require complementary loss-of-function analyses.

## 5. Conclusions

In summary, previous studies have documented the roles of OsMBL1 in biotic stress, abiotic stress responses, and leaf senescence. Our study further verified that *OsMBL1* is induced by various stresses, facilitates seed germination, and is correlated with stress tolerance through its association with altered expression of genes involved in biosynthesis and modification of flavonoids and changes in antioxidant systems, which expands our understanding of the potential role of *OsMBL1* in stress adaptation. Based on these observations, we propose a working model (Figure 5) in which *OsMBL1* overexpression leads to the constitutive upregulation of genes in the phenylpropanoid and flavonoid pathways, which is correlated with increased flavonoid accumulation and higher antioxidant enzyme activities, changes that are associated with improved stress tolerance. This model integrates our findings with previously reported roles of OsMBL1 in stress signaling. Nevertheless, this model is largely inferred from correlative data, and the critical molecular mechanisms underlying this regulatory cascade remain poorly understood. Devoid of canonical transcription factor domains, OsMBL1 presumably influences the transcription of metabolic genes through intermediate regulatory partners, yet these interacting mediators have not yet been identified. Direct evidence for a causal role of *OsMBL1* in regulating these pathways, and for the proposed enhancement of ROS scavenging capacity, will require targeted functional studies including loss-of-function mutants and direct ROS measurements under stress conditions.

## Data Availability

The entire data presented in this study are available upon request from the corresponding author.

## Supplementary Materials

The following supporting information can be downloaded at: https://www.mdpi.com/article/10.3390/biology15161378/s1, Figure S1. Expression patterns of *OsMBL1*. Transcript levels of *OsMBL1* were analyzed following treatments with NaCl (150 mM), PEG (10%), ABA (50 μM), H_2_O_2_ (10 mM), SNP (100 μM) and CaCl_2_ (10 mM), respectively. Leaf samples were collected at 0.5, 1, 6 and 12 h, as indicated. Data are presented as mean ± SD of three technical replicates from one independent experiment. Asterisks denote significant differences based on one-way ANOVA followed by Tukey’s multiple comparisons test; Figure S2. Functional verification of salt tolerance in *OsMBL1*-overexpressing transgenic rice. (A) Growth performance of WT and *OsMBL1*-overexpressing plants under normal growth conditions. (B) Phenotypes of WT and *OsMBL1*-overexpressing plants after 10 days of 150 mM NaCl stress treatment. (C) Phenotypes of WT and *OsMBL1*-overexpressing plants after 7 days of recovery following salt stress. (D) Survival rates of WT and *OsMBL1*-overexpressing plants after 7 days of recovery. Data are mean ± SD (n ≥ 3). Different asterisks indicate significant differences determined by Student’s *t*-test (* *p* < 0.05; ** *p* < 0.01); Table S1. Primers used in this study. Table S2. OsMBL1-regulating differentially expressed genes (DEGs) identified by RNA-seq. Table S3. Gene Ontology (GO) analysis of OsMBL1-regulating differentially expressed genes (DEGs). Table S4. Gene Ontology (GO) analysis of OsMBL1-regulating differentially expressed genes (DEGs).
