# Supplementary material for: Overexpression of OsMBL1 Is Associated with Changes in Flavonoid Biosynthesis and Antioxidant Capacity in Rice
Source: Biology (Basel). 2026 Aug 12;15(16):1378. doi: 10.3390/biology15161378 (PMC13509832; doi:10.3390/biology15161378)
Supplement: Supplementary file 1 [file biology-15-01378-s001.zip › Supplementary Figures.pdf]

Supplementary Figures

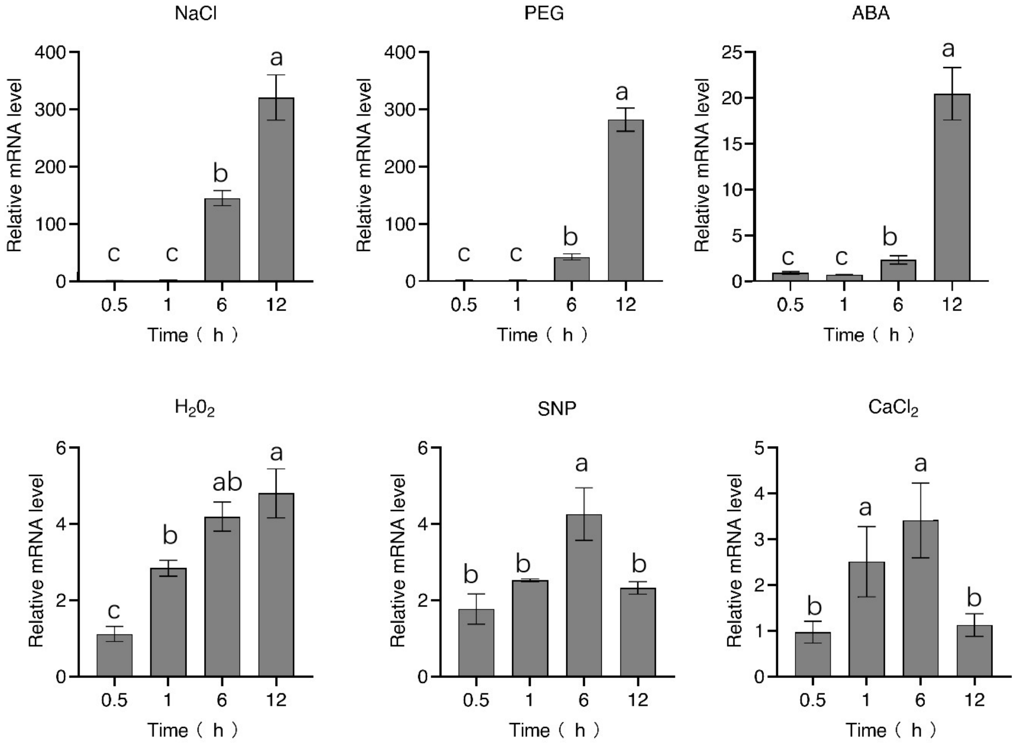

Figure S1. Expression patterns of *OsMBL1*. Transcript levels of *OsMBL1* were analyzed following treatments with NaCl (150 mM), PEG (10%), ABA (50 μM), H_2O_2 (10 mM), SNP (100 μM) and $CaCl_2$ (10 mM), respectively. Leaf samples were collected at 0.5, 1, 6 and 12 h, as indicated. Data are presented as mean ±SD of three technical replicates from one independent experiment. Asterisks denote significant differences based on one-way ANOVA followed by Tukey's multiple comparisons test.

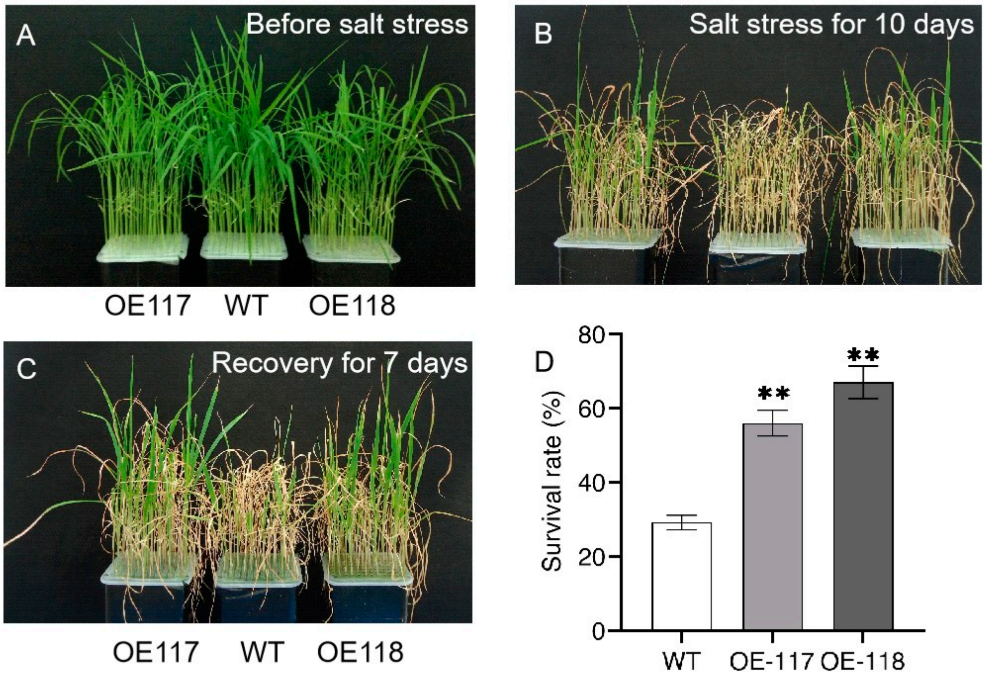

Figure S2. Functional verification of salt tolerance in *OsMBL1*-overexpressing transgenic rice. (A) Growth performance of WT and *OsMBL1*-overexpressing plants under normal growth conditions. (B) Phenotypes of WT and *OsMBL1*-overexpressing plants after 10 days of 150 mM NaCl stress treatment. (C) Phenotypes of WT and *OsMBL*1-overexpressing plants after 7 days of recovery following salt stress. (D) Survival rates of WT and *OsMBL1*-overexpressing plants after 7 days of recovery. Data are mean ± SD (n ≥ 3). Different asterisks indicate significant differences determined by Student's *t*-test (*$P < 0.05$; **$P < 0.01$).
